# Long Noncoding RNA X-Inactive-Specific Transcript Promotes the Secretion of Inflammatory Cytokines in LPS Stimulated Astrocyte Cell Via Sponging miR-29c-3p and Regulating Nuclear Factor of Activated T cell 5 Expression

**DOI:** 10.3389/fendo.2021.573143

**Published:** 2021-03-12

**Authors:** Mengqi Zhang, Haojun Yang, Zhuohui Chen, Xinhang Hu, Tong Wu, Weiping Liu

**Affiliations:** Department of Neurology, Xiangya Hospital, Central South University, Changsha, China

**Keywords:** lncRNA, microRNA, inflammatory cytokine, epilepsy, astrocyte

## Abstract

**Background:**

Astrocyte activation promotes glutamate accumulation and secretion of inflammatory factors, mainly responsible for epilepsy. Long noncoding RNA (lncRNA) X-inactive-specific transcript (XIST) regulates inflammation; however, the biological role and regulatory mechanism of XIST during astrocyte activation remain unclear.

**Methods:**

In the present study, rat epilepsy model and lipopolysaccharide (LPS)-treated CTX-TNA2 were established. XIST and miR-29c-3p expression were evaluated using quantitative real-time polymerase chain reaction. Nuclear factor of activated T cells 5 (NFAT5) was measured using western blot analysis. Interleukin (IL)-1β, IL-6, tumor necrosis factor-α, and L-glutamate levels in the culture supernatants were assessed using enzyme-linked immunosorbent assay. The binding between XIST and miR-29c-3p and between miR-29c-3p and the 3′-UTR of NFAT5 was analyzed using dual-luciferase reporter, RNA-binding protein immunoprecipitation (RIP), and Biotin pull-down assay. The proliferation and apoptosis were evaluated using CCK8 and flow cytometry, respectively.

**Results:**

XIST expression and NFAT5 protein level was increased, whereas miR-29c-3p expression was decreased in the epilepsy rat model and LPS-treated CTX-TNA2 cells. Silenced XIST expression, miR-29c-3p overexpression, or silenced NFAT5 expression inhibited the secretion of IL-1β, IL-6, and TNF-α and promoted glutamate transport in LPS-treated CTX-TNA2 cells. miR-29c-3p was the potential miRNA sponged by XIST. NFAT5 acted as a direct binding target of miR-29c-3p. Silenced miR-29c-3p expression or NFAT5 overexpression reversed the effect of silenced XIST expression on LPS-treated CTX-TNA2.XIST and miR-29c-3p treatment does not affect NFAT5 mRNA expression, but affects NFAT5 protein level. Furthermore, underexpressed XIST or overexpressed miR-29c-3p in LPS-stimulated CTX-TNA2 can attenuate neuronal apoptosis induced by LPS-stimulated CTX-TNA2.

**Conclusion:**

LncRNA XIST promotes the secretion of inflammatory cytokines in LPS- treated CTX-TNA2 *via* sponging miR-29c-3p and regulating NFAT5 expression.

## Introduction

Epilepsy is a common chronic neurological disorder occurring worldwide, which poses patients insomnia, depression, and memory and mood impairments and the economic burden on patients, their families, and the society (1). Epilepsy is caused by increased electrical discharges of abnormal neurons and presents typical clinical features, including recurrent disturbance, loss of consciousness, and uncontrolled muscle spasms and convulsions. Treatment over a long period in approximately 30% of patients with epilepsy will incur refractory and pharmacoresistant epilepsy (2). Numerous studies have ascertained that inflammatory dysregulation enormously contributes to the pathogenesis of epilepsy (3, 4). These studies reported that proinflammatory cytokines, interleukin-1β (IL-1β), IL-2, IL-6, and tumor necrosis factor- α (TNF-α) were significantly upregulated in the hippocampus and cerebrospinal fluid after epilepsy.

Astrocytes are the resident innate immune cells in the brain that provide trophic support to neurons, regulate glutamate transport and phagocytosis connectivity of neural circuits, control blood flow, and maintain brain homeostasis and blood–brain barrier (5). Moreover, they are one of the crucial glial cell types that produce numerous proinflammatory cytokines in the central nervous system during brain inflammation induced by central nervous system injury and disease (6). Activated astrocytes lose their functions, such as providing trophic support to neurons and maintaining brain homeostasis and blood–brain barrier (5). Epileptogenesis is associated with astrocyte activation and persistent inflammatory microenvironment in the neural tissue (7). Furthermore, inflammatory cytokines contribute to the loss of extracellular K+ and glutamate homeostasis and promote the release of gliotransmitters, such as glutamate-activating neuronal glutamate receptors, thereby affecting neuronal function. These changes in the microenvironment collectively have an impact on the hyperexcitability of the neuronal network and reduce the seizure threshold (3). Thus, astrocyte activation promotes glutamate accumulation and secretion of inflammatory factors, which play a pivotal role in the occurrence of epilepsy. Thus, exploring the molecular mechanism underlying astrocyte activation and secretion of inflammatory factors in epilepsy will help in understanding the development of epilepsy and in finding a novel therapeutic target for the disease.

Long noncoding RNAs (lncRNAs) lack protein-coding capacity and are longer than 200 nucleotides in length. At present, several studies have demonstrated that lncRNAs are key modulators in epilepsy (8, 9). Previous studies found that increased lncRNA ILF3-AS1 levels were observed in the hippocampus and serum of patients with temporal lobe epilepsy, which promoted inflammatory cytokine and MMP expression by targeting miR-212 (10). LncRNA NEAT1 promoted IL-6, COX-2, and TNF-α expression by targeting miR-129-5p and activating the Notch signaling pathway in epilepsy (11). LncRNA UCA1 was downregulated in epilepsy, whereas the UCA1/miR-203/MEF2C axis inhibited the activation of NF-κB signaling pathway and IL-6, TNF-α, and Cox-2 levels in epilepsy (12). lncRNA CASC2 suppressed the activation of astrocytes to reduce the frequency of epileptic seizures in epileptic rats, and CASC2 overexpression promoted PTEN expression to inhibit the release of adenosine and astrocyte activation (13). LncRNA X-inactive-specific transcript (XIST), which is a lncRNA located on the X chromosome, has been studied in various diseases to regulate inflammation. XIST triggered cell apoptosis and inflammatory cytokine levels in lipopolysaccharide (LPS)-induced injury in pneumonia by sponging miR-370-3p and upregulating TLR4 expression (14). XIST overexpression enhanced the inflammation level and promoted the epithelial–mesenchymal transition and infiltration of macrophages in the colonic tissue, thereby accelerating colorectal tumorigenesis and progression by sponging miR-133a-3p (15). XIST inhibition can suppress the inflammatory cytokine levels by hindering the activation of dorsal root ganglion satellite glial cells to alleviate pain and increase mechanical pain threshold in an inflammatory pain model (16); however, the biological roles and regulatory mechanisms of XIST during astrocyte activation in epilepsy remain unclear.

LPS-stimulated astrocytes can be present during inflammatory response (promote IL-1β, IL-6, and TNF-α expression) and be used to mimic epilepsy-inflammation to assess the underlying molecular or cellular mechanisms (17–19). In the present study, we used the LPS-treated CTX-TNA2, a rat astrocyte cell line, to mimic epilepsy-inflammation. First, we investigated the expression and biological roles of XIST in LPS-treated CTX-TNA2 cells. Furthermore, we studied the regulatory mechanism of XIST by sponging miRNA and regulating the target genes of miRNA. Our finding suggests that XIST can be used to improve astrocyte inflammation.

## Materials and Methods

### Rat Epilepsy Model

In total, 18 male Sprague Dawley (SD) rats (250–280 g, 6–8 weeks) were purchased from Southern Medical University (License Number: SCXK(YUE)2016-0041; Guangzhou, China). They were housed in an environment with 22 ± 2°C temperature and 50%–60% humidity under 12-h light/dark cycle. All SD rats had free access to food and water. All animal procedures were in accordance with those of National Institutes of Health Guidelines for the Care and Use of Laboratory Animals and were approved by the Animal Committee of Xiangya Hospital, Central South University. All SD rats were randomly divided into three groups (*n* = 6): normal, sham, and epilepsy groups. The epilepsy model was established, as previously described (13). Briefly, the epileptic rats were administered an intraperitoneal injection of 60 mg/kg pentylenetetrazol daily for 10 days. If rats reached grade IV–V according to the Racine criteria, the epilepsy models were considered as established (20). Rats in the sham group were injected with an equal amount of physiological saline instead of pentylenetetrazol. Rats in the control group received no treatment. On the 10th day after establishing the epilepsy model, all rats were euthanized through intravenous administration of pentobarbital (120 mg/kg). Subsequently, the dorsal hippocampus was collected from the rat brains for XIST and miRNA expression analysis.

### Cell Culture and Transfection

CTX-TNA2 (CRL-2006) astrocyte cell line was obtained from the American Type Culture Collection and was cultured in DMEM complete medium with 100 U/mL penicillin, 100 μg/mL streptomycin, and 10% fetal bovine serum (Gibco, Carlsbad, CA, USA). The medium was changed every 3 days. Thereafter, three siRNAs of XIST, si-NC, miR-29c-3p mimic, NC mimic, miR-29c-3p inhibitor, and NC inhibitor were transfected into the CTX-TNA2 according to experimental design. The transfection efficiency was assessed *via* quantitative real-time polymerase chain reaction (qRT-PCR). The siRNA sequence (GenePharma, Shanghai, China) was as follows: si-XIST-1, GGAAUGAGAUAAUGCUUAACG; si-XIST-2, GAAAUUGUAUACAAGAUUAAU; si-XIST-3, GGAUCUAAUCAUUCCUUUAAG; si-NC, UUCUCCGAACGUGUCACGUTT; miR-29c-3p mimic, ACCGAUUUCAAAUGGUGCUAUU; NC mimic, UCACAACCUCCUAGAGGAGAGAAA; miR-29c-3p inhibitor, UAACCGAUUUCAAAUGGUGCUA; and NC inhibitor, UCACAACCUCCUAGAGGAGAGAAA. Transfected cells were treated with or without LPS (1 µg/mL) for 24 h. Normal cultured (without LPS treatment) CTX-TNA2 acts as control group.

### qRT-PCR

The total RNA was extracted from the CTX-TNA2 using Trizol reagent (Invitrogen, Carlsbad, CA, USA). cDNA synthesis was performed using the ImProm-II™ Reverse Transcription System (Promega, Madison, WI, USA), according to the manufacturer’s protocol: 30°C 10 min, 42°C 60 min, and 85°C 10 min. Gene expression was evaluated *via* qRT-PCR using SYBR GREEN qPCR Super Mix (Invitrogen) on an ABI 7500 RT-PCR system (Applied Biosystems). GAPDH and U6 were used as the internal controls to calibrate the XIST, and U6 was used as the internal control for miR-29c-3p. The relative expression level data were analyzed using the 2^−ΔΔCt^ method. All reactions were performed in triplicates. The sequences of the primers used in this study were produced by Sangon Biotech (Shanghai, China) and presented as follows: XIST –F, 5′-GTCGTTCCTCACACCAGTCT-3′; XIST-R, 5′-CATCTGTCTTCCACTTTGGGC-3′, GAPDH-F, 5′-TGGGGCCAAAAGGGTCATCA-3′; GAPDH-R, 5′-GCAGGATGCATTGCTGACAA-3′, miR-29c-3p-F, 5′-ACACTCCAGCTGGGUAGCACCAUUUGAAAU-3′; miR-29c-3p-R, 5′-CTCAACTGGTGTCGTGGA-3′; U6-F, 5′-CTCGCTTCGGCAGCACATATACTA-3′; and U6-R, 5′-ACGAATTTGCGTGTCATCCTTGCG-3′.

### Enzyme-Linked Immunosorbent Assay

CTX-TNA2 culture supernatants were collected, and thereafter, IL-1β, IL-6, and TNF-αlevels in the culture supernatants were measured using the IL-1 Beta Rat enzyme-linked immunosorbent assay (ELISA) Kit (Invitrogen, BMS630), IL-6 Rat ELISA Kit (Invitrogen, ERA31RB), and Rat TNF-alpha ELISA Kit (Invitrogen, ERA56RB). Furthermore, to analyze the glutamate transport function of CTX-TNA2, 200 μM L-glutamate was added into the culture supernatants. After 24-h treatment, the L-glutamate level of culture supernatants was evaluated using the L-glutamate assay kit (Solarbio, Beijing, China). Optical density (OD) values were measured at 450 nm using a Multiskan Mk3 microplate reader (Thermo Fisher, Waltham, MA, USA). Lower L-glutamate levels of culture supernatants indicated better glutamate transport function. All experiments were performed in triplicate.

### Western Blot

Nuclear factor of activated T cell 5 (NFAT5) expression was assessed using the western blot assay, as previously described (21). Briefly, CTX-TNA2 cells were harvested and lysed in RIPA buffer (Beyotime, Shanghai, China). The mixture supernatants were separated using sodium dodecyl sulfate-polyacrylamide gel electrophoresis on 10% gels and were then transferred onto polyvinylidene fluoride membranes. The membranes were incubated overnight with NFAT5 monoclonal antibody (dilution, 1:1000, ab137407, Abcam, San Diego, CA, USA) and GAPDH loading control monoclonal antibody (dilution, 1:10,000, MA5-15738, Ebioscience, San Diego, CA, USA). The bound antibodies were detected using horseradish peroxidase-conjugated goat anti-mouse/rabbit IgG secondary antibody (dilution, 1:10,000, G-21040/21234, Ebioscience) and visualized using the enhanced chemiluminescent reagent (PerkinElmer Life Sciences, MA, USA).

### Dual-Luciferase Reporter Assay

The XIST sponging miRNAs were predicted using LncBase v3 (22). The miRNAs regulating target gene 3′-UTR were predicted using miRWalk (23). The wild-type (WT) or mutant (MUT) XIST was synthesized by GENEWIZ (Suzhou, China). The 3′-UTR of NFAT5 had 3 binding sites with miR-29c-3p. The upstream and downstream 500bp of miR-29c-3p WT or MUT binding site (only containing one putative binding sites) was synthesized by GENEWIZ. The WT or MUT-XIST was cloned into the 3′-end of the firefly luciferase gene of psi-CHECK2 vector. Then, 30 ng of either WT or MUT-XIST plasmids were cotransfected with 50 nM of miR-29c-3p mimics or miR-29c-3p inhibitor into 293T cells. Furthermore, the WT or MUT 3′-UTR of NFAT5 was cloned into the 3′-end of the firefly luciferase gene of psi-CHECK2 vector. Then 30 ng of either WT or MUT 3′-UTR of NFAT5 plasmids were cotransfected with 50 nM of miR-29c-3p mimics into 293T cells. After 48 h, the luciferase activity was measured using a dual-luciferase assay kit (Promega). Renilla luciferase activity was normalized against firefly luciferase activity.

### RNA-Binding Protein Immunoprecipitation Assay and Biotin RNA Pull-Down Assay

The RNA-binding protein immunoprecipitation (RIP) assay was performed using the RNA Immunoprecipitation Kit (GENESEED, Guangzhou, China). Briefly, CTX-TNA2 cells were lysed by RIP lysis buffer and cell supernatants were incubated overnight at 4°C with primary antibody against Ago2 or normal rat IgG and protein A/G magnetic beads. Then the complex bound to the magnetic bead was eluted. DNA in the complex bound to the magnetic bead was remove by column purification. And the RNA was purified by column purification and analyzed by the RT-qPCR assay to measure XIST and miR-29c-3p expression. The pull-down assay was performed using the PureBinding RNA-Protein pull-down Kit (GENESEED, Guangzhou, China). Briefly, CTX-TNA2 cells were transfected with biotinylated XIST probe (Bio-XIST-probe) or a negative control probe (Bio-NC-probe). At 48** h** after transfection, cells were harvested for biotin-based pull-down assay, followed by RT-qPCR assay to determine XIST and miR-29c-3p level.

### CCK8 and Flow Cytometry

CTX-TNA2 and neurons were cocultured using Transwell. CTX-TNA2 was seeded in the upper chamber, whereas neurons were seeded in the lower chamber. After the 24-h coculture, the proliferation and apoptosis of neurons were analyzed. Cell proliferation was detected using the CCK8 assay kit (Sigma-Aldrich, St. Louis, MO, USA) at 48 h after transfection, and the OD values were measured at 450 nm using a Multiskan Mk3 microplate reader (Thermo Fisher). Cell apoptosis was analyzed using a PE Annexin V Apoptosis Detection Kit I (BD Biosciences, San Jose, CA, USA) *via* flow cytometer (BD Biosciences). All experiments were performed in triplicates.

### Statistical Analysis

The experimental data were analyzed using SPSS 21.0 statistical software (IBM, Inc.). One-way analysis of variance was performed to analyze the statistical difference between four groups followed by *post hoc* Tukey tests. Data were presented as means ± standard deviation, and results with *P*-value < 0.05 were considered as statistically significant.

## Results

### Increased XIST Expression in Epilepsy Rat Model and LPS-Treated CTX-TNA2 Cells

**Figure 1:**
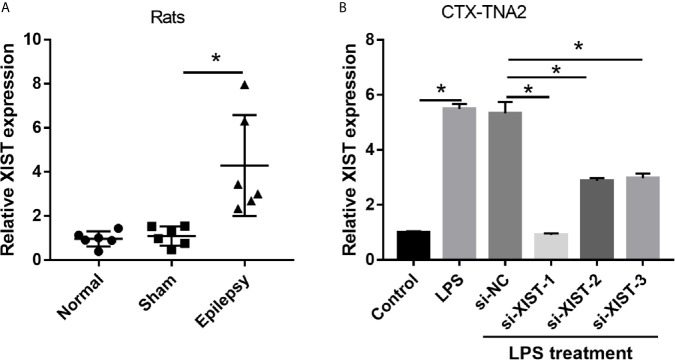
XIST expression was increased in LPS-treated CTX-TNA2 but was decreased after si-XIST transfection. **(A)** XIST expression was measured using qRT-PCR in epilepsy and in normal rat models. **(B)** XIST expression was measured using qRT-PCR in CTX-TNA2 treated with or without LPS (1 µg/mL) for 24 h, and it was measured using qRT-PCR in LPS-treated CTX-TNA2 after transfection of siRNAs at 24 h. *P < 0.05.

To explore the role of XIST in epilepsy, we assessed the XIST expression in epilepsy rat models and CTX-TNA2 cells treated with or without LPS. The RT-qPCR results revealed that XIST expression in epilepsy rat models was markedly higher than that observed in normal rats (**Figure 1A**). Moreover, we found that XIST expression in LPS-treated CTX-TNA2 was remarkably higher than that in CTX-TNA2 treated without LPS (**Figure 1B**).

### Silenced XIST Expression Inhibits the Secretion of Inflammatory Cytokines in LPS-Treated CTX-TNA2 Cells

**Figure 2:**
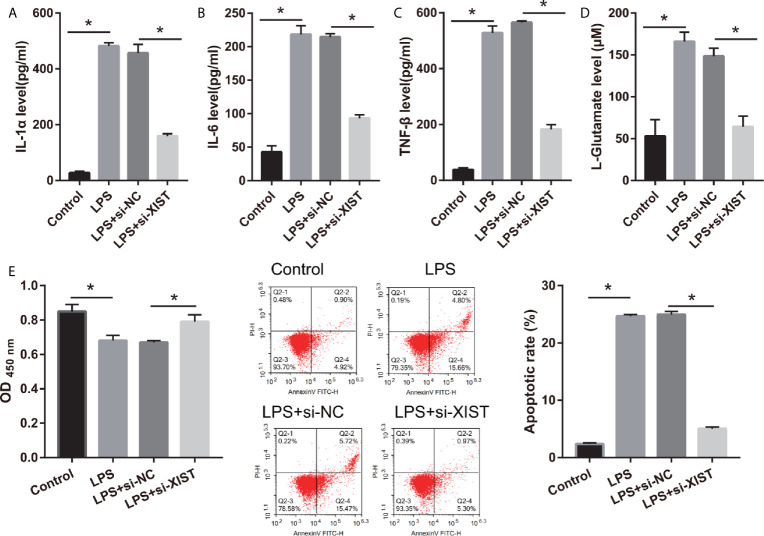
IL-1β, IL-6, TNF-α, L-glutamate levels, and neuronal apoptosis were decreased after si-XIST transfection in LPS-treated CTX-TNA2. **(A–D)** IL-1β, IL-6, TNF-α, and L-glutamate levels were measured using ELISA kit according to the standard procedures. **(E)** Neurons cocultured with normal cultured CTX-TNA2 (control group), LPS-treated CTX-TNA2 (LPS group), LPS-treated CTX-TNA2 transfected si-NC (si-NC group), and LPS-treated CTX-TNA2 transfected si-XIST (si-XIST group), respectively. The proliferation and apoptosis of neurons was assessed *via* CCK8 and flow cytometry, respectively, after coculturing with LPS-treated si-XIST-transfected CTX-TNA2 for 48 h. *P < 0.05.

To elucidate the biological roles of XIST in LPS-treated CTX-TNA2, we silenced XIST through siRNA transfection. We found that XIST expression was markedly reduced after transfecting three si-XISTs for 24 h, particularly si-XIST-1, based on the qRT-PCR results (**Figure 1B**). Thus, we selected si-XIST-1 (si-XIST) for further study. Moreover, we found that IL-1β, IL-6, and TNF-α levels, and L-glutamate levels in the culture supernatants were remarkably increased whereas glutamate transporter 1 (GLT1) and glutamate aspartate transporter 1 (GLAST) mRNA expression in CTX-TNA2 were remarkably decreased in LPS group compared with those in control group. Compared with LPS group and LPS+si-NC group, IL-1β, IL-6, and TNF-α levels, and L-glutamate levels in the culture supernatants were remarkably decreased whereas GLT1 and GLAST mRNA expression in CTX-TNA2 were remarkably increased in LPS+si-XIST group (**Figures 2A–D** and **Supplementary Figure 2**). Eventually, Neurons cocultured with CTX-TNA2. The results showed that neuronal proliferation was remarkably decreased and apoptosis was remarkably increased in LPS-treated CTX-TNA2 compared with that in the control group. Compared with LPS group and LPS+si-NC group, neuronal proliferation was remarkably increased and apoptosis was remarkably decreased in LPS+si-XIST group (**Figure 2E**). The results indicated that LPS stimulation leads to IL-1β, IL-6, TNF-α level increased, glutamate transporters GLT1 and GLAST expression and glutamate transport reduced, glutamate accumulation enhanced, and neuronal apoptosis, whereas inhibition of XIST expression restores CTX-TNA2 function.

### miR-29c-3p Reverses the Effect of XIST in LPS-Treated CTX-TNA2

**Figure 3:**
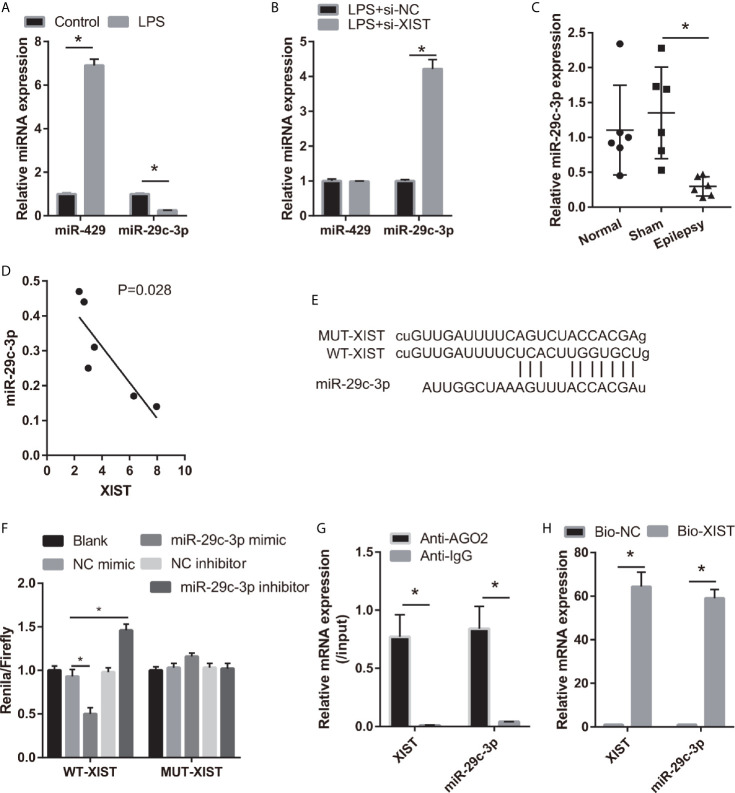
miR-29c-3p was the target miRNA of XIST. **(A)** miR-29c-3p expression was measured *via* qRT-PCR in CTX-TNA2 treated with or without LPS (1 µg/mL) for 24 h. **(B)** miR-29c-3p expression was measured *via* qRT-PCR in LPS-treated CTX-TNA2 after transfected siRNAs at 24 h. **(C)** miR-29c-3p expression was assessed *via* qRT-PCR in epilepsy rat models. **(D)** The correlation between XIST expression and miR-29c-3p expression was analyzed using Spearman correlation analysis. *P < 0.05. **(E)** The binding sites of XIST and miR-29c-3p. WT-XIST, Wild-type XIST sequence; MUT-XIST, mutant type XIST sequence of binding site. **(F)** The relative luciferase activity analysis after cotransfection with miR-29c-3p mimic or inhibitor and XIST WT or MUT (*n* = 3). **(G)** The expressions of miR-29c-3p and XIST were detected by qRT-PCR in CTX-TNA2 after Ago2 or IgG RIP assay. **(H)** The expressions of miR-29c-3p and XIST were detected by qRT-PCR in CTX-TNA2 after Biotin RNA pull-down assay.

To study the regulatory mechanism of XIST, we analyzed the potential miRNAs sponged by XIST *via* DIANA Tools (filter: brain tissue), and miR-29c-3p and miR-429 were found. The miR-29c-3p expression was lower and miR-429 expression was higher in the LPS-treated CTX-TNA2 than that in the control group (**Figure 3A**). Moreover, miR-29c-3p expression was higher; however, miR-429 expression did not change in the LPS-treated CTX-TNA2 after si-XIST transfection (**Figure 3B**). The miR-29c-3p expression was lower in epilepsy rat models than that in the sham group (**Figure 3C**). XIST expression in rats revealed a negative relationship with miR-29c-3p expression (**Figure 3D**). Thus, miR-29c-3p was the potential miRNA sponged by XIST. The binding sites of XIST and miR-29c-3p are illustrated in **Figure 3E**. Furthermore, the luciferase activity of the XIST-WT+ miR-29c-3p mimic group was markedly decreased compared with that in the XIST-WT+NC mimic group, whereas no difference was found between XIST-MUT+NC and XIST-MUT+ miR-29c-3p mimic groups (**Figure 3F**). RIP assay result showed that miR-29c-3p and XIST expression in the anti-AGO2 group was enriched, compared with that in the anti-IgG group (**Figure 3G**). Also, compared with Bio-NC group, miR-29c-3p and XIST expression was significant increase in Bio-XIST group (**Figure 3H**). These findings demonstrated that miR-29c-3p was a target of XIST in CTX-TNA2 cells. These results suggest that miR-29c-3p can bind with XIST.

**Figure 4:**
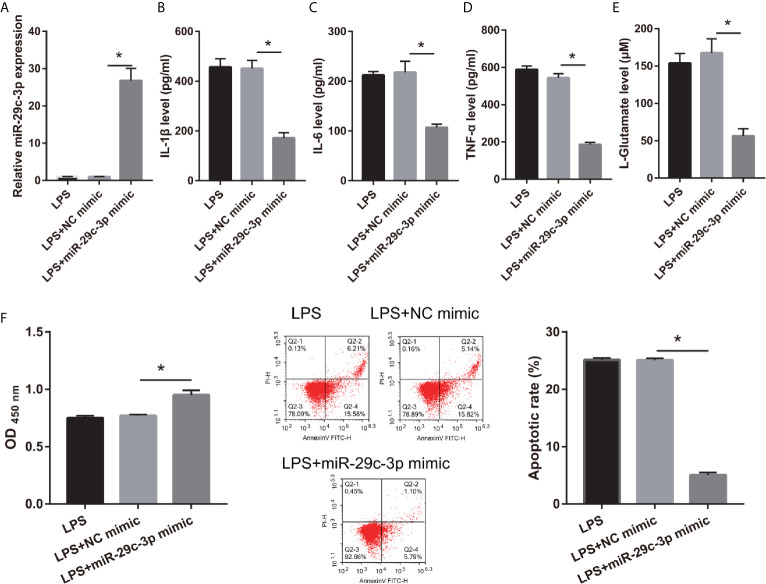
IL-1β, IL-6, TNF-α, L-glutamate levels, and neuronal apoptosis were decreased after miR-29c-3p mimic transfection in LPS-treated CTX-TNA2. **(A)** miR-29c-3p expression was measured *via* qRT-PCR after miR-29c-3p mimic transfection in LPS-treated CTX-TNA2 at 24 h. **(B–E)** IL-1β, IL-6, TNF-α, and L-glutamate levels were assessed using an assay kit according to the standard procedures. **(F)** Neurons cocultured with LPS-treated CTX-TNA2 (LPS group), LPS-treated CTX-TNA2 transfected NC mimic (NC mimic group), and LPS-treated CTX-TNA2 transfected miR-29c-3p mimic (miR-29c-3p mimic group), respectively. After cocultured at 48 h, the proliferation and apoptosis of neurons were evaluated *via* CCK8 and flow cytometry, respectively. *P < 0.05.

To assess the effect of miR-29c-3p in LPS-treated CTX-TNA2, miR-29c-3p expression was promoted by transfecting miR-29c-3p mimic. miR-29c-3p expression in LPS+miR-29c-3p group was significantly higher than that in LPS+NC mimic and LPS groups (**Figure 4A**). Additionally, XIST expression had no significant change between LPS, LPS+NC mimic, and LPS+miR-29c-3p mimic groups (**Supplementary Figure 1A**). Compared with the LPS+NC mimic group, IL-1β, IL-6, TNF-α, and L-glutamate levels in the culture supernatants were remarkably decreased whereas GLT1 and GLAST mRNA expression in CTX-TNA2 were remarkably increased in LPS+miR-29c-3p group (**Figures 4B–E** and **Supplementary Figure 2**). miR-29c-3p expression, IL-1β, IL-6, TNF-α, and L-glutamate levels, and GLT1 and GLAST mRNA expression had no obvious change between LPS and LPS+ NC mimic groups (**Figures 4B–E** and **Supplementary Figure 2**). Next, neurons cocultured with CTX-TNA2. The results showed that neuronal proliferation was remarkably increased and apoptosis was remarkably decreased in LPS+miR-29c-3p mimic group compared with that in the LPS+ NC mimic group. And neuronal proliferation and apoptosis had no obvious change between LPS and LPS+NC mimic groups (**Figure 4F**). The results indicated that overexpression miR-29c-3p inhibited IL-1β, IL-6, TNF-α levels, enhanced GLT1 and GLAST expression and glutamate transport, reduced glutamate accumulation, alleviate neuronal apoptosis, suggested that L miR-29c-3p overexpression relieved the effect of LPS on CTX-TNA2.

**Figure 5:**
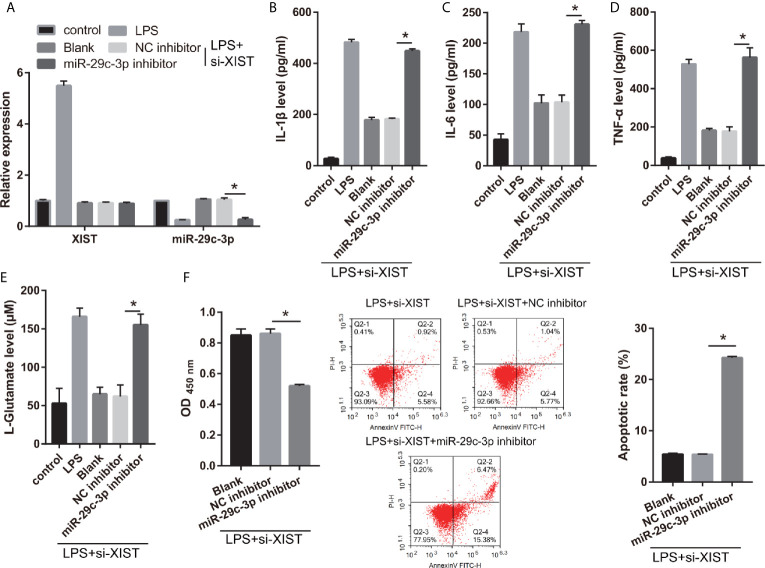
miR-29c-3p reverses the effect of XIST on LPS-treated CTX-TNA2. **(A)** XIST and miR-29c-3p expression were measured *via* qRT-PCR after si-XIST and miR-29c-3p mimic cotransfection in LPS-treated CTX-TNA2 at 24 h. **(B–E)** IL-1β, IL-6, TNF-α, and L-glutamate levels were assessed using an assay kit according to the standard procedures after si-XIST and miR-29c-3p mimic cotransfection in LPS-treated CTX-TNA2 at 24 h. **(F)** Neurons cocultured with LPS-treated si-XIST-CTX-TNA2 (LPS+si-XIST group), LPS-treated si-XIST-CTX-TNA2 transfected NC inhibitor (LPS+si-XIST+NC inhibitor group), and LPS-treated si-XIST-CTX-TNA2 transfected miR-29c-3p inhibitor (LPS+si-XIST+miR-29c-3p inhibitor group), respectively. After cocultured at 48 h, the proliferation and apoptosis of neurons were evaluated *via* CCK8 and flow cytometry, respectively. *P < 0.05.

Moreover, to further study the relationship between XIST and miR-29c-3p, the si-XIST (Blank group), si-XIST+NC inhibitor (NC inhibitor group), and si-XIST+miR-29c-3p inhibitor (miR-29c-3p inhibitor group) were transfected into CTX-TNA2 and then treated with LPS for 24 h. miR-29c-3p expression was remarkably decreased in miR-29c-3p inhibitor group than that in NC inhibitor group in LPS+ si-XIST-CTX-TNA2 (**Figure 5A**). And XIST expression in LPS+ si-XIST-CTX-TNA2 had no significant change between Blank, NC inhibitor, and miR-29c-3p inhibitor groups (**Supplementary Figure 1A**). Compared with the NC inhibitor group, IL-1β, IL-6, TNF-α, and L-glutamate levels in the culture supernatants were remarkably increased whereas GLT1 and GLAST mRNA expression in LPS+ si-XIST CTX-TNA2 were remarkably decreased in miR-29c-3p inhibitor group. And miR-29c-3p expression, IL-1β, IL-6, TNF-α, and L-glutamate levels, and GLT1 and GLAST mRNA expression in LPS+ si-XIST CTX-TNA2 had no obvious change between Blank and NC inhibitor groups (**Figures 5B–E** and **Supplementary Figure 2**). Furthermore, we cocultured neurons and LPS+ si-XIST CTX-TNA2. The result found that neuronal proliferation was markedly decreased and neuronal apoptosis was markedly increased in miR-29c-3p inhibitor group than that in NC inhibitor group (**Figure 5F**). And neuronal proliferation and apoptosis had no obvious change between Blank group and NC inhibitor groups. These results revealed that miR-29c-3p downexpression reversed the effect of XIST on LPS-treated CTX-TNA2.

### NFAT5 Acts as a Direct Binding Target for miR-29c-3p

**Figure 6:**
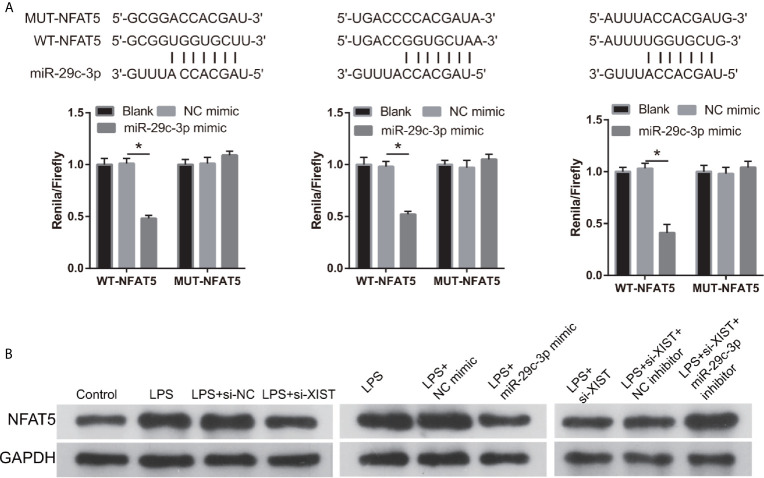
NFAT5 was a direct binding target of miR-29c-3p. **(A)** The binding sites of NFAT5 and miR-29c-3p. WT-XIST, wild-type NFAT5 sequence; MUT-XIST, mutant type NFAT5 sequence of binding site. The relative luciferase activity analysis after cotransfection with miR-29c-3p mimic or inhibitor and NFAT5 WT or MUT (*n* = 3). *P < 0.05. **(B)** NFAT5 expression was evaluated *via* western blot analysis after transfection at 24 h.

We found that NFAT5 may be potentially targeted by miR-29c-3p *via* miRWalk. The three binding sites of NFAT5 and miR-29c-3p are illustrated in **Figure 6A**. Moreover, the luciferase activity of the NFAT5-WT+ miR-29c-3p mimic group was remarkably decreased compared to that in the NFAT5-MUT+NC mimic group, whereas no difference was observed between the NFAT5-MUT+NC and NFAT5-WT+ miR-29c-3p mimic groups (**Figure 6A**). This means that all three sites can bind with NFAT5 3’-UTR, and the effect of three sites may be redundant or cooperative to controls NFAT5 protein levels. Additionally, NFAT5 protein level was markedly increased in LPS-treated CTX-TNA2 compared with that in non-LPS-treated CTX-TNA2, whereas the increased levels were reversed by transfection of si- XIST and miR-29c-3p mimic (**Figure 6B**). Furthermore, compared with si-XIST and si-XIST+NC inhibitor groups, NFAT5 protein level was markedly increased in the si-XIST+miR-29c-3p inhibitor group (**Figure 6B**). Finally, NFAT5 mRNA expression had no significant change after transfected si-XIST, miR-29c-3p mimic, and si-XIST+miR-29c-3p inhibitor (**Supplementary Figure 1C**). This result suggests that XIST and miR-29c-3p treatment does not affect NFAT5 mRNA expression, but affects NFAT5 protein level

### NFAT5 Reverses the Effect of XIST in LPS-Treated CTX-TNA2

**Figure 7:**
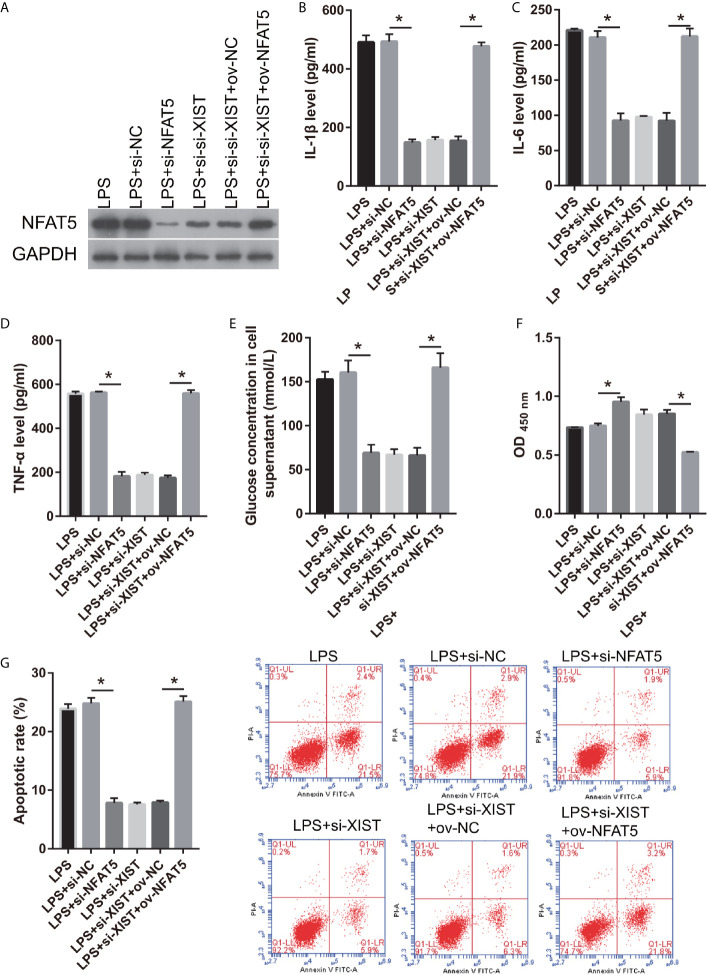
NFAT5 reverses the effect of XIST on LPS-treated CTX-TNA2. **(A)** NFAT5 expression were measured *via* western blot after transfection in LPS-treated CTX-TNA2 at 24 h. **(B–E)** IL-1β, IL-6, TNF-α, and L-glutamate levels were assessed using an assay kit according to the standard procedures after transfection in LPS-treated CTX-TNA2 at 24 h. **(F, G)** Neurons cocultured with LPS-treated CTX-TNA2 (LPS group), LPS-treated CTX-TNA2 transfected si-NC (LPS+si-NC group), and LPS-treated CTX-TNA2 transfected si-NFAT5 (LPS+si- NFAT5 group), LPS-treated si-XIST-CTX-TNA2 (LPS+si-XIST group), LPS-treated si-XIST-CTX-TNA2 transfected ov-NC (LPS+si-XIST+ov-NC group), and LPS-treated si-XIST-CTX-TNA2 transfected ov-NFAT5 (LPS+si-XIST+ ov-NFAT5 group), respectively. After cocultured at 48 h, the proliferation **(F)** and apoptosis **(G)** of neurons were evaluated *via* CCK8 and flow cytometry, respectively. *P < 0.05.

Moreover, the relationship between XIST and NFAT5 was further study. First, we found that NFAT5 expression was obviously reduced after transfected si- NFAT5 compared with transfected si-NC (**Figure 7A**). IL-1β, IL-6, TNF-α, and L-glutamate levels were markedly decreased in the si- NFAT5 group than that in si-NC group in LPS treated CTX-TNA2 (**Figures 7B–E**). And GLT1 and GLAST mRNA expression was remarkably increased in the si- NFAT5 group than that in si-NC group in LPS treated CTX-TNA2 (**Supplementary Figure 2**). Additionally, neuronal proliferation was markedly decreased and neuronal apoptosis was markedly increased in si-NFAT5 group than that in si-NC group after cocultured neurons and LPS treated CTX-TNA2 at 48 h (**Figures 7F, G**). The results showed that silenced NFAT5 expression can inhibit IL-1β, IL-6, TNF-α level, enhance glutamate transporters GLT1 and GLAST expression and glutamate transport, reduce glutamate accumulation, and alleviate neuronal apoptosis. Additionally, silenced NFAT5 expression have similar effects with silenced XIST

We also found that NFAT5 expression was obviously enhanced after transfected ov-NFAT5 compared with transfected ov-NC (**Figure 7A**). IL-1β, IL-6, TNF-α, and L-glutamate levels were markedly increased in the ov-NFAT5 group than that in ov-NC group in LPS treated si-XIST-CTX-TNA2 (**Figures 7B–E**). And GLT1 and GLAST mRNA expression was remarkably decreased in the ov-NFAT5 group than that in ov-NC group in LPS treated si-XIST-CTX-TNA2 (**Supplementary Figure 2**). Additionally, neuronal proliferation was markedly increased and neuronal apoptosis was markedly increased in ov-NFAT5 group than that in ov-NC group after cocultured neurons and LPS treated si-XIST-CTX-TNA2 at 48 h (**Figures 7F, G**). The results showed that overexpression NFAT5 expression can increased IL-1β, IL-6, TNF-α level, reduced glutamate transporters GLT1 and GLAST expression and glutamate transport, enhance glutamate accumulation, and increase neuronal apoptosis, which can reverse can reverse the protective effect of silencing XIST on LPS-treated cells.

## Discussion

Epilepsy is a neurological disorder that causes imbalance of inhibitory and excitatory neurons in the CNS (24). The activation of astrocytes and inflammation are associated with epileptogenesis and its frequency in patients with epilepsy, which may further result in damage or necrosis of brain neurons (12, 25, 26). Astrocytes have specific glutamate transporters include GLT1 and GLAST are responsible for glutamate uptake and maintain normal levels of glutamate in the extracellular space (27). Decreased glutamate transport has been observed in epileptic specimens (28). Dysregulation of astrocytic glutamate transporters, GLT-1 and GLAST, can impair astrocytic glutamate clearance and enhance glutamate accumulation which also leads to increased neuronal discharge and excitation and induction of epilepsy, which could lead to novel glial therapeutics for epilepsy (29). Additionally, overactive astrocytes stimulate proinflammatory cytokine release and have been shown to play a crucial role to neuronal loss following inflammatory stimulation (30). Excessive inflammatory in the astrocytes inihibits glutamate transport and promotes neuronal hyper-excitability, contributed to the generation of seizures (31, 32). LPS is a prototypical endotoxin that induces an inflammatory response in astrocytes and is used to investigate neuroinflammatory diseases. Peripheral injection of LPS can accelerate epileptogenesis or enhance epilepsy-induced damage in animal models (33, 34). Previous study also found that LPS-induced astrocytes act as neurological diseases model such as epilepsy, stroke, or traumatic brain injury (19, 35). In this study, we found that LPS treatment can reduce GLT1 and GLAST expression and enhance inflammation and glutamate accumulation in astrocytes lead to neuronal apoptosis, which similar with the effect of astrocytes in epilepsy.

In this study, XIST expression was found to be significantly up-regulated in the rat epilepsy model. XIST plays a pivotal role in the of progression of various inflammation diseases. XIST was overexpressed in patients presenting spinal cord trauma with injured astrocytes, and play an active regulatory role in the dysfunction module involved in the biological processes of inflammation, oxidation, and apoptosis (36). XIST aggravated the inflammatory cell infiltration and degree of fibrosis in LPS-induced acute respiratory distress syndrome in mice (37). Moreover, inhibition of XIST reduced GFAP, IL-6, and TNF-α expression; suppressed satellite glial cell activation; and ameliorated inflammatory pain (16). These studies revealed that XIST can regulate LPS-induced inflammation. XIST can regulate disease progression by sponging miRNAs (36, 37). miRNAs widely participate in various neurodegenerative diseases, such as epilepsy (38, 39). In the present study, we found that miR-29c-3p was the potential miRNA sponged by XIST. miR-29c-3p expression was found to be significantly reduced in the rat epilepsy model, which had negative relationship with XIST expression. miR-29c-3p had functional implications in the physiological and pathological processes. The expression of miR-29c-3p, which acts as a biomarker, was reduced in patients with Alzheimer’s disease (40). Moreover, the overexpression of miR-29c-3p suppressed proinflammatory cytokine release in LPS-stimulated BV-2 cells with anti-inflammatory properties (21), revealed that miR-29c-3p can inhibit LPS-induced inflammation. In accordance with the aforementioned studies, we also found that XIST expression was enhanced and miR-29c-3p expression was reduced in LPS-treated CTX-TNA2 cells.

NFAT5, a new member of the Rel family, has an ambivalent effect under various pathological conditions. NFAT5 expression in astrocytes of injured hippocampi was promoted after inducing brain edema and excitotoxic neuronal death (41). Increased NFAT5 expression induced by inflammatory signals aggravated blood–brain barrier injury, neuroinflammation, and neuron hyperexcitability-induced epilepsy (42). In contrast, *NFAT5* overexpression protected the astrocytes against ischemic damages and inhibited neuron hyperexcitability-induced seizures (43). In this study, NFAT5 expression was increased in LPS-stimulated CTX-TNA2, which was in accordance with results of a previous study (42). Furthermore, we found that NFAT5 acted as the target gene of miR-29c-3p. NFAT5 protein expression was inhibited by silencing XIST or overexpressing miR-29c-3p expression. Additionally, silenced NFAT5 expression have similar effects with silenced XIST, and overexpression NFAT5 can reverse the effect of XIST on LPS-stimulated CTX-TNA2 cells. These results suggested that miR-29c-3p, sponged by XIST, inhibited NFAT5 to silence astrocyte activation and ameliorate inflammatory-induced neuronal apoptosis.

This study has certain limitations. First, XIST, NFAT5, and miR-29c-3p expression was not verify in patients with SHR-AF. Furthermore, XIST, NFAT5, and miR-29c-3p effect on epilepsy rat model were not elucidate. Additionally, this study found that XIST can adsorb miR-29c-3p. It was also found that XIST can regulate the expression of miR-29c-3p. However, the mechanism by which XIST regulates the expression of miR-29c-3p remains unclear.

## Conclusion

XIST and NFAT5 expression was increased while miR-29c-3p expression was decreased in epilepsy rat models and LPS-stimulated CTX-TNA2 cells. Additionally, silenced XIST inhibited the secretion of inflammatory cytokines and promoted glutamate transport in LPS-stimulated CTX-TNA2 by sponging miR-29c-3p and regulating NFAT5 expression, which can attenuate neuronal apoptosis. These findings indicated that XIST and miR-29c-3p were the novel therapeutic targets that impede astrocyte activation, which might provide a new strategy for the treatment of epilepsy.

## Data Availability Statement

The raw data supporting the conclusions of this article will be made available by the authors, without undue reservation.

## Ethics Statement

All animal procedures were in line with National Institutes of Health Guidelines for the Care and Use of Laboratory Animals and approved by the Animal Committee of Xiangya Hospital, Central South University.

## Author Contributions

MZ designed the study, collected and analyzed the data, and wrote the manuscript. HY and ZC collected the data. XH and TW analyzed the data. WL designed the study and revised the manuscript. All authors read and approved the final manuscript. All authors contributed to the article and approved the submitted version.

## Funding

This study was supported by the National Natural Science Foundation of China (no. 81501025), the Natural Science Foundations of Hunan Province (nos. 2020JJ4893 and 2020JJ4134), and the Key Research and Development Program of Hunan Province (no. 2020SK2063). The funding organization did not contribute in the design, execution, analysis, interpretation, or presentation of this work.

## Conflict of Interest

The authors declare that the research was conducted in the absence of any commercial or financial relationships that could be construed as a potential conflict of interest.

## Supplementary Material

Supplementary Figure 1 XIST, miR-29-3p, and NFAT5 RNA levels had no significant change between 24 h transfection and 48 h transfection groups in CTX-TNA2 with and without LPS stimulation. XIST **(A)**, miR-29-3p **(B)**, and NFAT5 **(C)** RNAs levels was measured by qRT-PCR in CTX-TNA2 with and without LPS stimulation after transfection at 24 h and 48 h.

Supplementary Figure 2 XIST, miR-29-3p, and NFAT5 regulated glutamate transporter 1 (GLT1) and glutamate aspartate transporter 1 (GLAST) mRNA expression in CTX-TNA2. GLT1 **(A)** and GLAST **(B)** expression was measured using qRT-PCR in CTX-TNA2 treated with or without LPS (1 µg/mL) after transfection at 24 h.
