# Supplementary figures and images for: Long Noncoding RNA X-Inactive-Specific Transcript Promotes the Secretion of Inflammatory Cytokines in LPS Stimulated Astrocyte Cell Via Sponging miR-29c-3p and Regulating Nuclear Factor of Activated T cell 5 Expression
Source: Front Endocrinol (Lausanne). 2021 Mar 12;12:573143. doi: 10.3389/fendo.2021.573143 (PMC7995889; doi:10.3389/fendo.2021.573143)

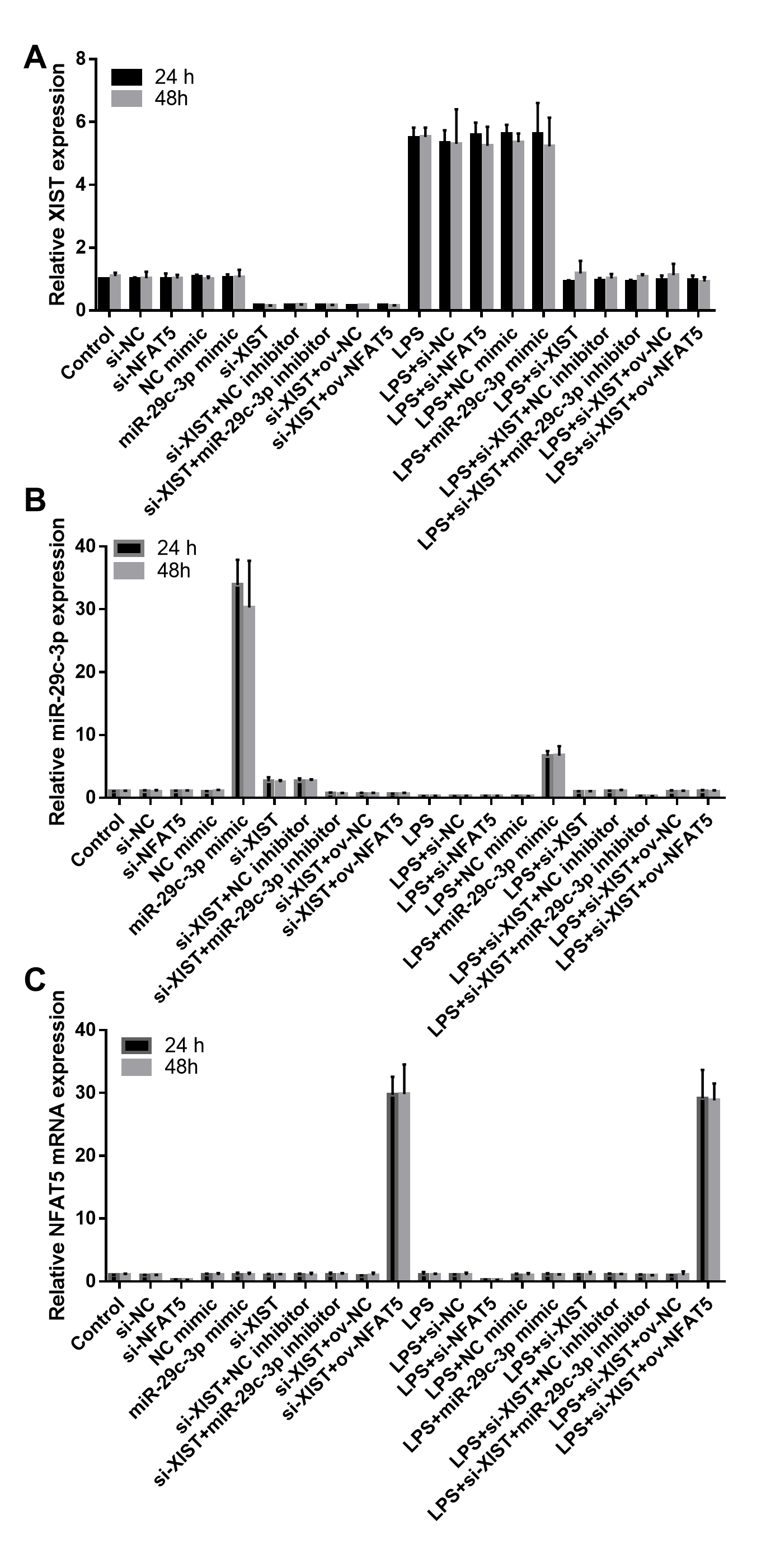

Supplement: Supplementary Figure 1 — XIST, miR-29-3p, and NFAT5 RNA levels had no significant change between 24 h transfection and 48 h transfection groups in CTX-TNA2 with and without LPS stimulation. XIST (A), miR-29-3p (B), and NFAT5 (C) RNAs levels was measured by qRT-PCR in CTX-TNA2 with and without LPS stimulation after transfection at 24 h and 48 h. [file Image_1.tif]

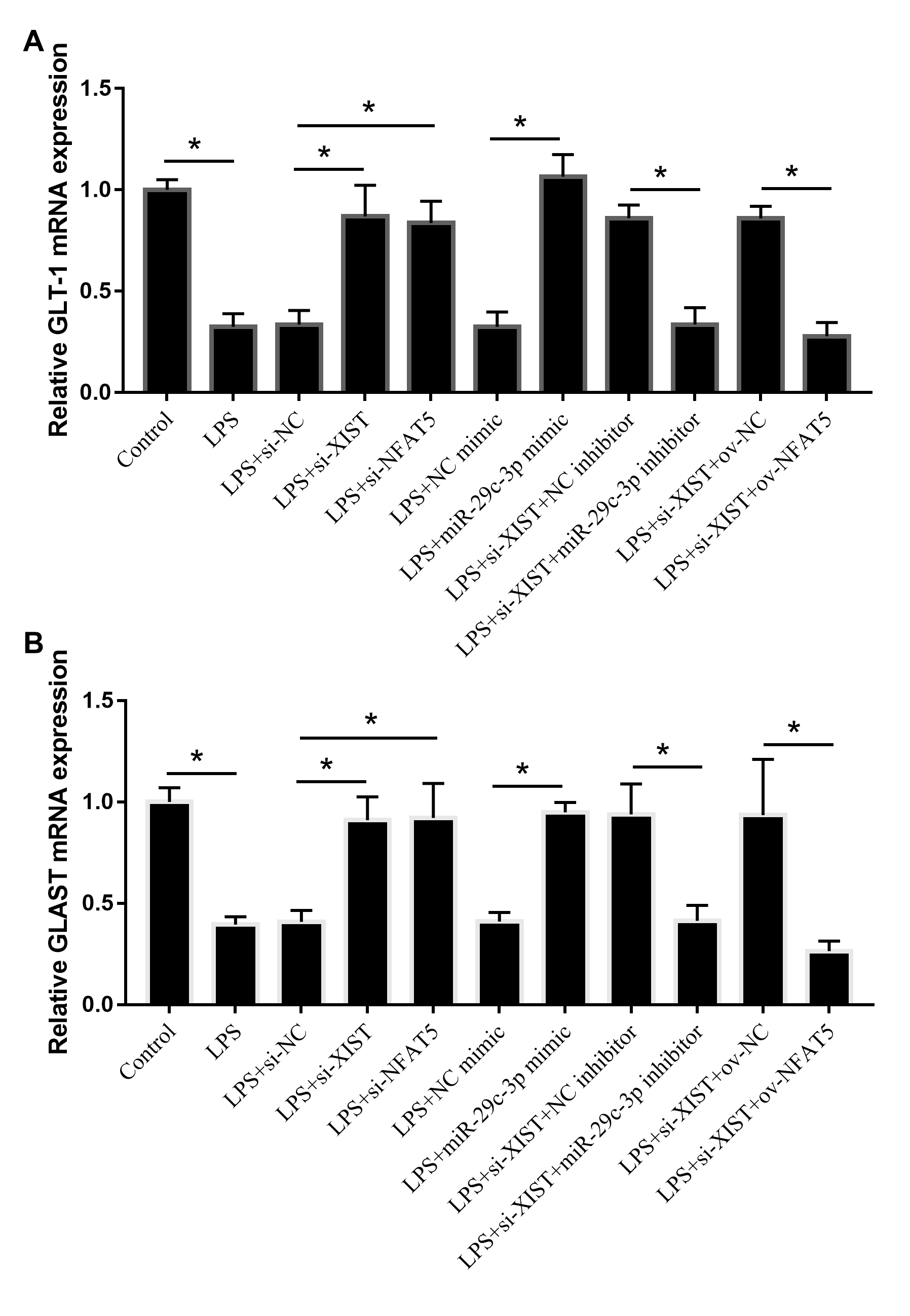

Supplement: Supplementary Figure 2 — XIST, miR-29-3p, and NFAT5 regulated glutamate transporter 1 (GLT1) and glutamate aspartate transporter 1 (GLAST) mRNA expression in CTX-TNA2. GLT1 (A) and GLAST (B) expression was measured using qRT-PCR in CTX-TNA2 treated with or without LPS (1 µg/mL) after transfection at 24 h. [file Image_2.tif]
